# Developing standardized patient-based cases for communication training: lessons learned from training residents to communicate diagnostic uncertainty

**DOI:** 10.1186/s41077-021-00176-y

**Published:** 2021-07-22

**Authors:** Dimitrios Papanagnou, Matthew R. Klein, Xiao Chi Zhang, Kenzie A. Cameron, Amanda Doty, Danielle M. McCarthy, Kristin L. Rising, David H. Salzman

**Affiliations:** 1 Department of Emergency Medicine, Sidney Kimmel Medical College at Thomas Jefferson University, 1025 Walnut Street, College Building, Suite 100, Room 101, Philadelphia, PA 19107 USA; 2 Department of Emergency Medicine, Northwestern University Feinberg School of Medicine, Chicago, IL USA; 3 Division of General Internal Medicine and Geriatrics/Department of Medicine and Department of Medical Education, Northwestern University Feinberg School of Medicine, Chicago, IL USA; 4 Department of Emergency Medicine, Thomas Jefferson University, Philadelphia, PA USA; 5 Department of Medical Education, Northwestern University Feinberg School of Medicine, Chicago, IL USA

**Keywords:** Diagnostic uncertainty, Communication training, Standardized patients, Case design

## Abstract

Health professions education has benefitted from standardized patient (SP) programs to develop and refine communication and interpersonal skills in trainees. Effective case design is essential to ensure an SP encounter successfully meets learning objectives that are focused on communication skills. Creative, well-designed case scenarios offer learners the opportunity to engage in complex patient encounters, while challenging them to address the personal and emotional contexts in which their patients are situated. Therefore, prior to considering the practical execution of the patient encounter, educators will first need a clear and structured strategy for writing, organizing, and developing cases. The authors reflect on lessons learned in developing standardized patient-based cases to train learners to communicate to patients during times of diagnostic uncertainty, and provide suggestions to develop a set of simulation cases that are both standardized and diverse. Key steps and workflow processes that can assist educators with case design are introduced. The authors review the need to increase awareness of and mitigate existing norms and implicit biases, while maximizing variation in patient diversity. Opportunities to leverage the breadth of emotional dispositions of the SP and the affective domain of a clinical encounter are also discussed as a means to guide future case development and maximize the value of a case for its respective learning outcomes.

**Supplementary Information:**

The online version contains supplementary material available at 10.1186/s41077-021-00176-y.

## Introduction

In 1999, the Accreditation Council for Graduate Medical Education (ACGME) mandated that all medical residency graduates achieve competency in interpersonal and communication skills by the completion of training [1]. Since then, significant curricular development has focused on teaching and refining communication and interpersonal skills in trainees [2]. Today, trainees in the health professions are immersed in experiential exercises that support effective relationship building, sharing of medical information, decision-making, exploring next steps in treatment plans, and paying attention to patients’ emotions [2]. In addition, the focus on effective communication now extends beyond undergraduate and graduate medical education, involving program accreditation and maintenance of certification for providers already in practice [3].

Human simulation programs, in which human role players interact with learners in contexts that support both learning and assessment, remain a cornerstone for communication training [4]. Standardized or simulated patients (SPs) are individuals who are trained to appropriately convey characteristics of specific patients in an encounter, and are routinely employed by programs for this purpose [5, 6]. SPs can be actors, lay-persons, real patients (i.e., former patients with the lived experiences of being a patient, or patients with a chronic, but not disabling, disease), or clinicians who are trained to follow predefined scenarios and provide standardized responses to learners’ behaviors from the perspective of many different roles [5, 7]; for this reason, the term *simulated participant* has been recently used as a more inclusive term to represent breadth of human role players in the context of simulation [4].

SPs can be trained to provide learners with valuable feedback on their behaviors and attitudes, as well as their verbal and nonverbal communication skills [8]. Although the terms standardized patient and simulated patient are often used interchangeably, the context in which SPs are working will determine the standardization (i.e., consistency and reliability) of their behavior [4]. In the context of high stakes (i.e., summative) assessment, for example, where SPs will need to be behave in a standardized manner [4], case design and development are critical in ensuring that learning objectives surrounding communication skills are met.

Well-designed case scenarios offer learners the opportunity to engage in complex SP encounters, and challenge them to synthesize care delivery within the personal and emotional contexts in which their patients are situated [9]. Talwankar et al. share best practices on how to run an effective standardized patient scenario. The authors highlight the importance of maximizing simulated scenarios by focusing on quintessential parameters of the session, including facilitator and SP training, technology, debriefing, and engagement of all involved stakeholders [4, 9–11].

Before being able to facilitate an SP encounter, however, educators first need an intentional strategy for case design and development. Detailed attention to case development is important for a single case, and is particularly relevant if developing multiple encounters (e.g., to ensure for broad representation of a specific topic or for different iterations of an assessment).

**Fig. 1:**
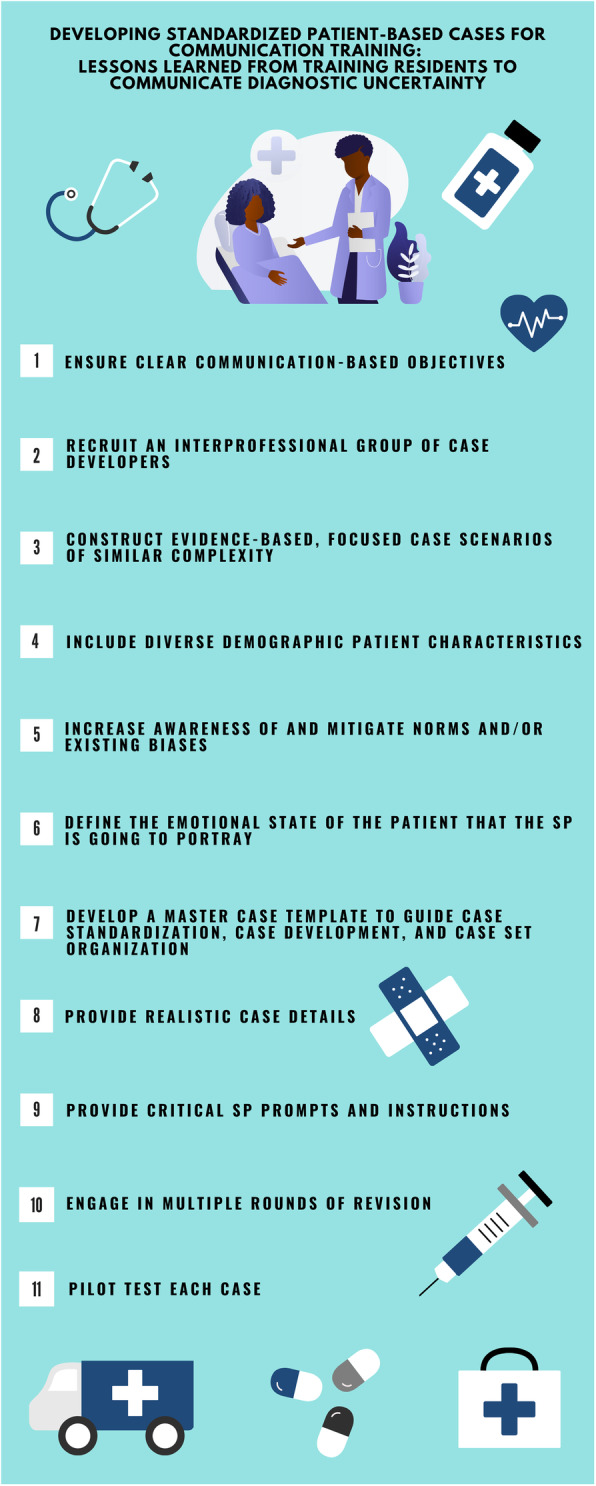
Developing standardized patient-based cases for communication training: lessons learned from training residents to communicate diagnostic uncertainty

In this article, we share lessons learned from iteratively developing and curating a set of simulation cases for communication-based training in our graduate medical education SP program (Fig. 1) [12]. Specifically, our simulation program focused on educating trainees on how to improve the quality of their communication with patients who are discharged from the emergency department (ED) without a pathologic diagnosis [12]. Despite the frequency of diagnostic uncertainty in acute care settings, there is minimal training on how to safely and effectively transition ED patients home in the setting of uncertainty [13]. After developing the Uncertainty Communication Checklist [12], our team designed and developed SP-based cases for communication training during times of diagnostic uncertainty in the ED. We describe our process, which resulted in a set of cases that is both standardized (with respect to complexity and the communication skill being assessed) and diverse (with respect to the standardized patients’ demographics and emotional presentation).

As our program was geared toward formal assessment of these communication skills in our resident physicians based on the Uncertainty Communication Checklist [12], we explicitly discuss the design and development of cases for standardized patients. Our intention was to build a series of cases in a structured way that would allow for ample opportunities for formal assessment (i.e., for use in objective structured clinical examinations, OSCEs) [5]. Since variation in role play was not required in our program, we opted not to leverage simulated patients at the present time. We understand that our experiences will be more relevant to North American audiences than to audiences in Europe and Asia, where simulated patients are far more used than standardized patients [5]. In addition to discussing key steps and workflow processes that can assist educators with case set design, we review the need to increase awareness of and mitigate existing norms and biases, while maximizing the variation in case diversity and complexity. We also describe opportunities to leverage the emotional dispositions of the SP not only to guide case development but also to curate a collection of cases that represent a wide breadth of personas that SPs can portray.

## Ensure clear communication-based objectives

Simulation is an effective tool for teaching and improving communication skills [14]. As with any educational intervention, successful communication-based simulation requires well-designed and targeted learning objectives. Both the INACSL (International Nursing Association for Clinical Simulation and Learning) Standards of Best Practice: Simulation^SM^ and the ASPE (Association of Standardized Patient Educators) Standards of Best Practice highlight the importance of clear communication-based objectives when designing simulations in general and when working with SPs [4, 10, 11]. Scenarios must be deliberately constructed to facilitate these objectives. Given that our intervention aimed to address training on how to communicate diagnostic uncertainty, our cases explicitly focused on communication skills and avoided extraneous cognitive or psychomotor tasks.

The purpose of the simulation should be clearly articulated at the beginning of the session, and the learner’s communication task (e.g., breaking bad news, death notification, patient handoff, discharge discussions) should be stated explicitly. To ensure an appropriate focus on the case’s communication objective(s) and reduce unnecessary distractions, learners should be assured that all clinical information provided (i.e., patient history, imaging, laboratory results) are factually accurate and does not demand re-interpretation.

Careful consideration and description of the learner’s role and environment can also significantly influence communication strategies through constructive alignment [15]. For example, a goals-of-care conversation during a scheduled outpatient visit will significantly differ from a similar conversation in the emergency department (ED), where a patient is seriously ill [16]. The environment in which the simulation takes place should be selected intentionally and be congruent with the learning objectives of the case.

## Recruit an interprofessional group of case developers

The importance of organizing a team with diverse professional backgrounds to develop and write your communication training cases cannot be overstated. A diverse, interprofessional team can support more robust case development, implementation, and evaluation of the case, the case scenario, and the case learning objectives [17]. Such a team is likely to create a more credible and realistic case narrative. Furthermore, communication itself is a complex process to compartmentalize into a discrete learning experience. Communication is replete with cultural and linguistic differences between providers, patients, and their families [18]. The added dimension of a patient’s culture (i.e., ethnicity, race, nationality, religion) may prompt additional misunderstanding if the case scenario addresses sensitive cultural issues [18]. In designing our set of cases for communication training, we composed a development team that consisted of physicians, content experts (i.e., communication scientists), simulation educators, and interprofessional education (IPE) champions.

## Construct evidence-based, focused case scenarios of similar complexity

Constructing a series of varied clinical scenarios of similar complexity is essential for the success of communication-based simulation [10, 11, 19]. For example, a curriculum for sharing bad news with a patient should encompass a range of serious diagnoses for practice and assessment [20]. This practice allows learners to develop familiarity with the broader communication framework (e.g., breaking bad news, in general) as opposed to an individual application (e.g., revealing a diagnosis of lung cancer, specifically). While the content of the cases should vary, the complexity should remain similar across scenarios. For example, a diagnosis of a non-operative minor fracture would not be equivalent to a terminal illness when breaking bad news. Even though communication skills occur within the context of the management of a patient, attention must be given to ensure that learning objectives focus on a communication skill as opposed to the management of an individual clinical scenario.

Cases should also be constructed to reflect plausible scenarios that learners are likely to encounter in clinical practice. Authors may utilize published literature or publicly available data sets, such as the National Hospital Ambulatory Medical Care Survey [21], to ensure that cases reflect the array of diagnoses encountered in various practice settings. Clinical scenarios also must be appropriate for the designated communication task. For our simulations, which focused on communicating diagnostic uncertainty with ED patients at the time of discharge, we included diagnoses that were appropriate for discharge (e.g., uncomplicated diverticulitis, and not unstable angina). We consulted with leadership in clinical informatics to verify common diagnoses that were discharged from the ED with symptom-based final diagnoses. To further support realism in these SP-based simulations, cases designed should incorporate current treatment guidelines reflecting evidence-based practices [22].

## Include diverse demographic patient characteristics

Communication-based simulation education should include cases constructed to reflect the diversity of patients encountered in clinical practice. Incorporating these elements into simulation requires deliberate effort on the part of educators. A review of simulation technology identified a lack of diverse characteristics in mannequins and task trainers, particularly in the areas of skin tone, age, and weight [23]. For communication-based simulation, which may rely more on interpersonal interactions than technology, recruitment of a diverse pool of SPs is essential [24]. We recognize that diversity of certain features, such as age, ethnicity, and gender expression, depends largely on the SPs available. Case authors should ensure that other forms of diversity, including sexual orientation, health literacy, and socioeconomic status, are also reflected in the case design. After developing vignettes and scenarios for our cases based on communication-oriented objectives, we adapted each of these cases to represent a wide range of patient-specific demographics. We then worked closely with our simulation center’s SP program to recruit a diverse pool of SPs for our training program.

## Increase awareness of and mitigate norms and/or existing biases

During case creation and curriculum development, medical educators must recognize the potential impact of implicit bias, defined as the “unconscious, unintentional assumptions people make” [25]. Due to the unconscious nature, people are not aware of the bias and the unintentional influence on their behavior [26]. One of the core principles of implicit bias is the automatic cognitive process that exists to allow for creation of heuristics to improve the speed of interpretation of new situations, at the risk of accuracy [27]. Traditionally, heuristics have been used in medical education as a means to teach pattern recognition. Backhus et al. describe that “stereotypes of diseases are one of the cornerstones of medical education and allow us to acquire and synthesize a large volume of information and expeditiously arrive at a diagnosis and treatment plan” [28].

While pattern recognition may increase efficiency of learning, this approach inadvertently enforces patterns based on implicit bias, which are detrimental to our learners and patients. The impact implicit bias has on contributing to the disparities in care have been described across several domains including communication, treating patients’ pain, recommendation for thrombolysis or arthroplasty, and diagnosis of COPD [29].

In designing cases and developing patient personas, it is critical to understand the impact that unconscious bias may have on case development, and take active steps to mitigate the perpetuation of stereotypes that influence disparities in patient care. Identification of one’s own implicit biases through completion of an Implicit Association Test may help educators reflect on their awareness of potential biases, and help bring them into consciousness during case design [30]. Careful attention should be made by educators to avoid reinforcing common stereotypes associated with patient names, race, ethnicity, gender, sexual orientation, age, religion, socio-economic status, native language, and citizenship. Lewis et al. recommends ensuring that “cases are based on authentic problems and respect the individuals represented in a case to avoid bias or stereotyping marginalized populations” [4]. If cases are successfully designed with these concepts in mind, learners are less likely to be triggered by specific case prompts (e.g., age, symptoms) and avoid reflexively generating narrow differential diagnoses and pursuing anticipated management plans [28]. In the early stages of case design and development for our training program, the entire team carefully reviewed cases for existing stereotypes, triggers, prompts. Prior to the start of the program, cases underwent several rounds of iterative review by the development team. Thus, a deliberate approach to case generation in the context of recognizing our own implicit biases may help reduce the unintended propagation of stereotypes for learners, and may result in more equitable and unbiased communication with all of our patients.

## Define the emotional state of the patient that the SP is going to portray

Incorporating challenging, yet authentic, SP emotional states in communication-based simulations requires detailed planning and careful documentation to ensure that a wide range of emotional states are represented in case design and development [31]. For learners to be successful in a communication-based scenario, they must be able to develop rapport with the patient portrayed by the SP; for these cases, developing and assessing interpersonal skills are quintessential. To add, this layer of authenticity to case development requires that learners fully engage with the emotions of their SPs. Case developers should clearly define the possible emotional states available for the SP to assume for a specific case; this task should be prioritized along with scripting other essential parameters of the case [32]. Case developers should consider the range of realistic emotional states that are congruent with the clinical context of the specific case being developed (i.e., from reassured to anxious) (Table 1). A detailed explanation of these emotional states is also included in Additional files (Additional file 1). From a case design perspective, each emotional state should prompt specific responses and behaviors from the learner, independent of other cues embedded in the case (Additional files 2 and 3). Selecting unique emotional states for a single medical case will easily maximize the educator’s case set, while diversifying the patient presentations learners are exposed to in the SP training program.

**Table 1:** Examples of emotional states an SP can take with sample instructions for the conversation

Emotional state	Case example	SP instructions for the conversation	Closing comments (only if needed)*
Reassured	The patient presented to the ED seeking reassurance about a specific diagnosis and has received it. The patient is receptive to the conversation. The patient is comfortable going home without a definite diagnosis. The patient asks reaffirming and clarifying questions throughout the scenario. When/if the physician indicates that no specific diagnosis has been found, the patient responds in a reassured manner.	• Greet provider upon entry. • Express that you are reassured about how you are physically feeling right now (i.e., you’re your symptoms are better and have not worsened). Share that you feel reassured with the results so far when they are disclosed to you as normal. Ask how the physician can explain your symptoms. When/if the physician indicates that no specific diagnosis has been found, you respond that you are nonetheless reassured. You can express your reassured state with the following example phrases during appropriate parts of the conversation: “So it doesn’t look like anything serious? That’s such good news.” “As long as we aren’t finding anything scary, I’m ok.” “Thank goodness. I was worried you’d find something terrible.”	“It’s ok, I am reassured by everything you have shared with me today. Thank you for your time and care.”
Confused	Throughout the encounter, the patient is confused about the lack of a diagnosis and the inability to find anything specifically wrong. The patient will focus on the lack of a diagnosis. When/if the physician indicates that no specific diagnosis has been found, the patient responds in a confused manner. Although the patient is confused, he/she does not display hostile or aggressive behavior.	Greet provider upon entry. Express eagerness to hear the results. Inquire with a confused nature, about what the results signify when they are disclosed to you as normal: “I just don’t understand. How can they be normal?” Ask how the physician can explain your symptoms. When/if the physician indicates that no specific diagnosis has been found, you respond in a confused manner. You can express your confusion with the following phrases during appropriate parts of the conversation: “I don’t understand how you don’t have an answer.” “That’s weird, how could everything be normal when I feel this way? Shouldn’t they show something?” “I wonder why this happened to me.”	“Thank you for trying to help me today. I’m still a bit confused that I don’t have a diagnosis for my symptoms, but I appreciate your time and explanation.”
Inquisitive Inquiring	The inquisitive/inquiring patient is genuinely interested in understanding what is going on and asks many questions. The patient has researched symptoms online, has spoken to friends, and is invested in his/her care. The patient is eager to learn about next steps. The patient asks probing questions based on information discloses. When/if the physician indicates that no specific diagnosis has been found, he/she responds in an inquiring manner. Questions are not aggressive, nor do they suggest incorrect management. Rather, questions represent curiosity with regards to care.	• Greet provider upon entry. • Ask inquiring questions regarding your symptoms. • Inquire with a curious nature about what the results signify when they are disclosed as normal: • “So, what do normal results mean?” • Ask how the physician can explain your symptoms. When/if the physician indicates that no specific diagnosis has been found, you respond in an inquiring manner. • You can express your curiosity with the following phrases during the conversation: o “I read online that this could be consistent with XXX, could that be the cause?” o “What else could be going on?” o “How do you know that I won’t develop worse symptoms?”	“I was hoping to get an answer today, but thank you for explaining things to me.” “ I am still curious about what is happening, but I’m all set to go home now.”
Nervous Anxious	The patient is nervous about his/her visit and is anxiously awaiting results. The patient becomes more nervous after not being provided with a diagnosis. The patient was initially scared to seek evaluation, as a family member was recently diagnosed with cancer. It took a lot of courage for the patient to seek care. The patient is worried about receiving his/her results, as he/she is terrified about receiving a “bad” diagnosis. No one has yet disclosed any results to the patient, which is making him/her even more nervous. When/if the physician indicates that no specific diagnosis has been found, the patient responds in a nervous manner. Although the patient is nervous, there is no hostile or aggressive behavior noted.	Greet provider upon entry. Express nervousness about getting results. Inquire, with a nervous/anxious nature, about what the results signify when they are disclosed to you as normal: “Are you sure they are normal?” “How can you be sure the tests are normal?” Ask how the physician can explain your symptoms. When/if the physician indicates that no specific diagnosis has been found, you respond in a nervous/anxious manner. You can express your nervousness with the following phrases during the conversation: “Are you sure I’m not dying?” “How do you know that I won’t develop worse symptoms?” “What if my symptoms are a sign of something really bad?”	“Thanks for trying to help me today.” “I’m still quite nervous, but I appreciate your time and explanation.”

*In the event the learner does not adequately respond to the patient’s emotional state that the SP is portraying in column 1, in addition to the SP prompts provided in column 2, the SP can then terminate the conversation using the closing statement(s) provided

Once the emotional states are selected, they should be evenly distributed and combined with other case descriptors (e.g., a 45-year-old male with a headache who is extremely anxious about his chief complaint, versus a 45-year-old female with a similar headache who is easily reassured during the visit about her chief complaint). Our team developed a color-coded system for use within a master grid (see tip 7) to ensure that emotional states and other key variables were fairly distributed across the case set. Although this process may represent a reductionist and formulaic approach to case development, it ensures that learners will be challenged to interact and communicate with patients portrayed by SPs with a wide range of emotional states and dispositions.

## Develop a master case template to guide case standardization, case development, and case set organization

Simulation case design requires a thoughtful and deliberate approach to ensure the case meets the specific goals and objectives for the session. When developing multiple scenarios, it is beneficial to start with the construction of a master case template to standardize the process and ensure the content created aligns with the overall objectives prior to individual case creation. Guidance for creation of a structure is supported by Lewis et al., who provide suggestions for creating scenarios using standardized patients [4]. The authors encourage development of a “simulation design that is repeatable” and contains “information for the SPs related to situation and backstory, history, affect and demeanor” [4]. Features of simulation-based education that support effective learning include ensuring clinical variation, when applicable, and allowing for learning to occur in a controlled environment [33]. A master case template will help ensure these recommendations are accounted for by the case developer. The template that we employed for our SP-based cases were similar to those described in the literature; however, we included a detailed section dedicated to the emotional state of the patient the SP was to portray to our learners.

We identified the need to have a template for case development during our work with training resident physicians on communicating diagnostic uncertainty at the time of ED discharge [12]. Our communication training program required the creation of simulated cases to afford learners the opportunity to demonstrate their ability to perform this communication task. To standardize the process of case development, we first identified key variables that varied from case to case. A patient characteristics grid (Table 2) was created, with each row representing a unique case scenario. Spreadsheet programs, such as Microsoft^TM^ Excel or Google^TM^ Spreadsheet, can be used to organize the variables for each case (e.g., SP’s emotional state and demographic characteristics). In our grid, columns represented patient characteristic variables that we modified in each case, which corresponded to specific components on the case template. A color-coded case template is included in Additional files (Additional file 4). These components included initial presenting chief complaint, patient name, gender, age, associated medical comorbidities, testing completed during the ED visit, primary patient encounter versus sign-out patient, ongoing versus resolved symptoms, and emotional state.

**Table 2:**
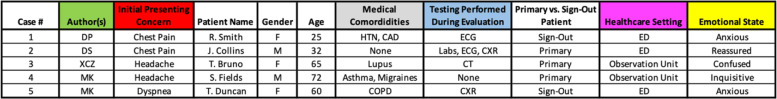
Patient characteristics grid with color-coded variables that can vary across cases

When creating multiple cases for a similar complaint (i.e., initial presenting concern), it is essential to ensure standardization and that each individual case contains all of the information necessary for the SP to portray the patient in a consistent manner. Thus, we created a case summary guide for the SP, which is included in Additional files (Additional file 3). The case summary guide contained multiple sections that could be modified based on information contained in the patient characteristic grid. Topics in the guide included the patient’s opening statement, a description of the patient’s emotional state, a brief synopsis of the case, instructions for the SP, specific questions permissible for the SP to ask during the conversation, and specific examples in the checklist pertaining to the patient’s chief complaint. Case components that needed to be modified for each patient scenario were color coded to match the patient characteristic grid. This strategy allowed multiple authors to simultaneously construct cases while maintaining consistency across the case set, and ensured that all essential components were included. Finally, a standardized file and case naming convention was applied to allow for rapid case identification for specific case components of interest.

## Provide realistic case details

Each case scenario should be content specific, clinically authentic, and based on current clinical practice [22, 34]. While technology-enhanced simulators have demonstrated encouraging effects on learning outcomes, there is simply no substitute for real human interaction when it comes to teaching and learning communication skills [23]. For this reason, careful case writing and realistic case design, that approximates case presentations from the clinical environment, are critical [35]. For simulations focused on communication skills, learners should be immersed in a case that provides them with adequate clinical information, typical to what they would be presented with in the clinical environment, to engage in a conversation with an SP. The history of present illness (HPI), past medical history (PMH), physical exam, and elements of the diagnostic workup should reflect the standard of care and evidence-based practices [36].

Given that learning objectives for these cases fall within the domain of interpersonal and communication skills, learners should not be distracted and/or derailed by cues that may be factually incorrect or confusing. For example, a case with a chief complaint of chest pain should include appropriate and consistent HPI and PMH elements that would prompt the learner to naturally consider the etiologies in question (i.e., acute coronary syndrome, pulmonary embolism, acute aortic dissection). Similarly, diagnostic results should be clearly labeled, with reference ranges, and presented as they would in the clinical environment (e.g., full radiology report with impression for a cardiac stress test) to both heighten case fidelity and mitigate cognitive load [37].

This “lesson learned” was discovered early in our experiences with case design and development. Our overarching goal for the training program was to engage residents with communicating diagnostic uncertainty to their SPs. To support these conversations, our residents required the clinical details to first ascertain the clinical course for their patients, to quickly come to the conclusion that their patients’ results were within normal limits, and to ultimately appreciate that no clinical diagnosis was reached during their patient’s visit to the ED. In the initial versions of our cases, full details on the patient’s clinical course (i.e., laboratory values, radiology interpretations) were unintentionally omitted from the case; in several instances, this prompted residents to reconsider medical management, rather than to fully engage in conversation with SPs about the lack of a diagnosis and address any patient concerns. Furthermore, initial versions of our cases also unintentionally excluded normal reference ranges for specific tests; interpretation of test results significantly distracted residents from the communication-based learning objectives of the case. Eventually, our team realized that inclusion of this data into each of the cases mitigated learner distraction and maximized their engagement in communicating diagnostic uncertainty to their SPs.

## Provide critical SP prompts and instructions

While emotional states of the SP will heighten the realism of communication-based simulations, case developers should be mindful of the effects these emotions may have on overall case narrative and flow [38]. Furthermore, because learner performance cannot be predicted, developers must consider all possible directions in which a case may evolve (i.e., if the learner does not pick-up on the SP’s situational emotional cues) and appropriately build-in scripted instructions to guide SP behaviors (i.e., when to terminate an encounter) [38]. Table 1 provides several examples of instructions and statements the SP can use to guide the conversation for specific emotional states, as well as closing comments the SP can use to end the encounter should the learner not pick-up on the emotional cues.

To address learners’ unexpected performance and better prepare SPs for their respective roles, case developers should agree on a descriptive profile for each emotional state. This description may include scripted responses the SP can make during the encounter, and provide detailed instructions to the SP on when to amplify or dampen a specific emotional state. Case developers may consider providing SPs with a preparatory, relatable, and empathetic story arc for each emotional state to assist them with achieving the right mindset for the upcoming case encounter [39]. Below is an example of a story arc we provided to one of our SPs who was instructed to convey an inquisitive emotional state to the learner, per the case’s learning objectives:

A patient with an ‘inquisitive’ emotional state is genuinely interested in understanding what is going on and asks many probing questions. This is the person who has researched their symptoms on the internet; has spoken to friends about his/her condition; and is greatly invested in his/her own care and is determined to know all the details about his/her condition(s) and what next steps should be.

Detailed scripts may assist SPs with case progression. SPs, however, should be encouraged to ask open-ended questions within the context of their respective emotional states, and avoid asking specific questions that could lead to prompting for checklist items. For our cases, the validated checklist our team developed to communicate diagnostic uncertainty (i.e., the Uncertainty Communication Checklist) [12], was shared with SPs. For each item on the checklist, our team provided detailed examples to SPs on what statements and open-ended questions they could make during the encounter. Similarly, for each checklist item, questions that could prompt learners for these items were highlighted and avoided by SPs. If the learner failed to progress through the checklist and the case, and did not appropriately respond to cues provided by the SP, case developers provided the SPs with closing comments [or what we refer to as the case ripcord] to end the scenario. For the emotional story arc example above, the SP portraying a patient with an inquisitive emotional state may conclude the case by stating:

I was really hoping to get an answer today. I appreciate you trying to explain things to me. I am still very curious about what is happening with me; but I’m all set to go home now.

## Engage in multiple rounds of revision

Cases should be iteratively revised with the entire team to ensure that each case appropriately corresponds to its respective learning objectives [40]. Revisions should not be rushed, as they are critical in standardizing scenarios where communication tasks are involved [41]. This process will help augment case set validity and reliability, which is especially important for high-stakes simulation scenarios (e.g., when cases are used for the summative evaluation of learners) [41]. Serial revisions during case design can help to attain the appropriate level of realism needed to maintain learner engagement and elicit behaviors that are most representative of learner performance in a specific clinical environment [40]. Details regarding the clinical environment and relevant scenario-related data should also be iteratively reviewed to maintain alignment with the intended conversation the learner is to have with the SP [40].

To facilitate multiple rounds of revision, each case being developed was shared with a sub-group of the team as a Google^TM^ document. The sub-group was responsible for drafting the case, cross-referencing clinical details of the case for accuracy, and making edits and comments. Upon completion of the case draft, the document was then shared with the entire team for review, and openly discussed at a team meeting. Edits and suggestions were tracked and applied to the case draft. This process was repeated several times until there was consensus on the case by all members of the case development team.

## Pilot test each case

After a new case has been written, reviewed, and revised by the writing team, the case should move on to piloting. At least one individual from the writing team should be present to record any recommendations or changes that need to be made to the case scenario. Ideally, participants undergoing the pilot simulation should be representative of the learner population for which the case was originally intended and be unfamiliar with the case documents (e.g., not a member of the team). Initial pilot testing, focused on refining elements of the SP script, can be accomplished with a member of the case design team serving as the SP. If available, review by an SP trainer may provide additional observations for refinement. It is highly advised that this pilot testing and revision process take place prior to SP training.

We also recommend that all required resources for the case are available in order to ensure that the scenario runs as realistically as possible [40]. To this effect, staff from the simulation center should be present if the case is intended to take place in a simulated environment. Additionally, a member of the technical team should be present during pilot testing if there is a desire to employ audiovisual systems (e.g., optimizing camera and microphone placement, if the session is to be recorded). Most importantly, piloting a newly developed scenario focused on communication skills will help determine if the case requires modification to better meet stated learning objectives [40]. Such continuous revisions will allow for the systematic refinement of simulation cases that can improve the overall quality of case design [42].

## Conclusion

Successful integration of simulation and SPs into communication skills training programs requires a clear strategy to thoughtfully write and organize cases. Educators will benefit from an organized workflow that allows for seamless tracking of patient characteristics, standardization of case complexity, and ease of future case development. A methodical approach to writing SP cases will help guide educators in the health professions maximize their success in developing a set of standardized, diverse cases focused on communication skills.

While our approach to case design and development is limited by specifically describing our own personal experiences developing cases for use in a communication-based program to teach learners how to navigate conversations surrounding diagnostic uncertainty, our approach does consider implicit bias, existing norms, and stereotypes, and leverages the emotional state of the patient portrayed by the SP to enrich the learning experience. By considering the breadth of possibilities that exist within the affective domain of a potential SP encounter (i.e., the range of emotions an SP can experience or the various demeanors an SP can take during an encounter), the more likely educators will be able to maximize the value of a case for its respective learning outcomes.

## Supplementary Information

**Additional File 1.** Emotional States.

**Additional File 2.** Example of an SP Encounter Template.

**Additional File 3.** Example of an SP Case Summary Guide.

**Additional File 4.** Color-Coded Case Template Corresponding to Key Patient Characteristics.

## Data Availability

Not applicable. No data is reported in the manuscript.
