# Supplementary material for: Developing standardized patient-based cases for communication training: lessons learned from training residents to communicate diagnostic uncertainty
Source: Adv Simul (Lond). 2021 Jul 22;6:26. doi: 10.1186/s41077-021-00176-y (PMC8296470; doi:10.1186/s41077-021-00176-y)
Supplement: Supplementary file 1 — Additional File 1. Emotional States. [file 41077_2021_176_MOESM1_ESM.docx]

**Additional File 1: Emotional States**

Note about emotional states: Emotional states should be considered a starting point for the conversation. If you start as [nervous/confused/inquiring], and the physician appropriately completes the checklist and addresses your emotional state, you should become progressively more reassured over the course of the interview. Specific instructions to guide the SP during the conversation are provided.

Instructions for when to employ the closing comment: Only use the closing comment provided if the physician is not appropriately progressing through the checklist and/or is not responding to prompts below.

Emotional State: **REASSURED**

The patient presented to the Emergency Department seeking reassurance about a specific pathologic diagnosis (i.e., cancer, stroke, heart attack) and has received it.

The patient is receptive, amenable to the conversation. The patient came seeking specific reassurance about something dangerous and is comfortable going home without a definite diagnosis. During the conversation, the patient asks reaffirming and clarifying questions throughout the scenario (i.e., “So you are saying that I do not have anything scary going on with me, right?”).

When/if the physician indicates that no specific diagnosis has been found, the patient responds in a reassured manner (i.e., “I feel so much better knowing this”).

Specific instructions for the SP for the conversation:

- Greet the physician upon entry into room.
- Express that you are reassured about how you are physically feeling right now (i.e., you’re your symptoms are better and have not worsened).
- Share that you feel reassured with the results so far when they are disclosed to you as normal.
- Ask how the physician can explain your symptoms.
- When/if the physician indicates that no specific diagnosis has been found, you respond that you are nonetheless reassured.
- You can express your reassured state with the following example phrases during appropriate parts of the conversation:
- “So it doesn’t look like anything serious? That’s such good news”
- “As long as we aren’t finding anything scary, I’m ok.”
- “Thank goodness. I was worried you’d find something terrible.”

Closing Comment (if needed): “It’s ok, I am reassured by everything you have shared with me today. Thank you for your time and care.”

Emotional State: **NERVOUS/ANXIOUS**

The patient is nervous about his/her visit to the Emergency Department and is anxiously awaiting results. The patient becomes more nervous after not being provided with a diagnosis for his/her chief complaint.

The patient was initially scared to present to the Emergency Department for evaluation because a family member of theirs recently received a cancer diagnosis. It took a lot of courage for the patient to come on. Additionally, the patient is worried about receiving his/her results. The patient is terrified about receiving a “bad” diagnosis. No one has yet disclosed any results to the patient, which is making him/her even more nervous.

When/if the physician indicates that no specific diagnosis has been found, the patient responds in a nervous manner (i.e., “Are you sure?”). Although the patient is nervous, there is no hostile or aggressive behavior noted.

Specific instructions for the SP for the conversation:

- Greet provider upon entry to the room.
- Express nervousness about getting results.
- Inquire with a nervous/anxious nature about what the results signify when they are disclosed to you as normal (i.e., “Are you sure they are normal? How can you be sure the tests are normal?”).
- Ask how the physician can explain your symptoms.
- When/if the physician indicates that no specific diagnosis has been found, you respond in a nervous/anxious manner.
- You can express your nervousness with the following phrases during appropriate parts of the conversation:
  - “Are you sure I’m not dying?”
  - “How do you know that I won’t develop worse symptoms?”
  - “What if my symptoms are a sign of something really bad?”

Closing Comment (if needed): “Thanks for trying to help me today. I’m still quite nervous, but I appreciate your time and explanation.”

Emotional State: **CONFUSED**

Throughout the encounter, the patient is confused about the lack of a diagnosis and the inability to find anything specifically wrong. The patient will focus on the lack of a diagnosis.

When/if the physician indicates that no specific diagnosis has been found, the patient responds in a confused manner (i.e., “I am confused. How could the testing not reveal an explanation for my symptoms?” or “It is odd that the tests did not reveal anything. how could that be?”). Although the patient is confused, he/she does not display hostile or aggressive behavior.

Specific instructions for the SP for the conversation:

- Greet provider upon entry to the room.
- Express eagerness to hear the results.
- Inquire with a confused nature, about what the results signify when they are disclosed to you as normal (i.e., “I just don’t understand. How can they be normal?”).
- Ask how the physician can explain your symptoms.
- When/if the physician indicates that no specific diagnosis has been found, you respond in a confused manner.
- You can express your confusion with the following phrases during appropriate parts of the conversation:
  - “I don’t understand how you don’t have an answer.”
  - “That’s weird, how could everything be normal when I feel this way? Shouldn’t they show something?”
  - “I wonder why this happened to me.”

Closing Comment (if needed): “Thank you for trying to help me today. I’m still a bit confused that I don’t have a diagnosis for my symptoms, but I appreciate your time and explanation.”

Emotional State: **INQUISITIVE/INQUIRING**

The inquisitive/inquiring patient is genuinely interested in understanding what is going on and asks many questions. The patient has researched his/her symptoms online, has spoken to friends, and is greatly invested in his/her own care. He/she is eager and interested to know what is going on and what should be done next. To this effect, the patients asks probing questions throughout the entire conversation based on what the provider discloses.

When/if the physician indicates that no specific diagnosis has been found, the patient responds in an inquiring manner (i.e., “Well, that’s interesting. How unexpected. I was sure you would know what is going on.”).

The questions are not overly aggressive and do not suggest that the physician has done anything incorrectly; rather, the patient’s questions represent curiosity with regards to his/her care.

Specific instructions for the SP for the conversation:

- Greet the physician upon entry into room.
- Ask inquiring questions regarding your symptoms.
- Inquire with a curious nature, about what the results signify when they are disclosed to you as normal (i.e., “So, what do normal results mean?”).
- Ask what the physician sees in your evaluation that can explain your symptoms.
- When/if the physician indicates that no specific diagnosis has been found, you respond in an inquiring manner.
- You can express your curiosity with the following phrases at appropriate parts of the conversation:
  - “I read online that this could be consistent with XXX, could that be the cause?”
  - “I wonder what else could be going on?”
  - “How do you know that I won’t develop worse symptoms?”

Closing Comment (if needed): “I was hoping to get an answer today, but thanks for explaining things to me. I am still curious about what is happening, but I’m all set to go home now.”
