# Supplementary material for: Developing standardized patient-based cases for communication training: lessons learned from training residents to communicate diagnostic uncertainty
Source: Adv Simul (Lond). 2021 Jul 22;6:26. doi: 10.1186/s41077-021-00176-y (PMC8296470; doi:10.1186/s41077-021-00176-y)
Supplement: Supplementary file 2 — Additional File 2. Example of an SP Encounter Template. [file 41077_2021_176_MOESM2_ESM.docx]

**Additional File 2: Example of an SP Encounter Template**

**Case Title:** Chest Pain/Male/Sign-out/Reassured

**Standardized Patient Name:** Tyler Miller

**Gender:** Male

**Age Range:** 43 years old

**Setting:** Emergency Department

**Primary vs. Sign-Out Patient:** Sign-out

**General Appearance/Dress:** Sitting on hospital recliner wearing hospital gown

**Emotional State:** Reassured

**Initial Presenting Symptoms:** Chest Pain

**Symptoms:** Resolved
