# Supplementary material for: Developing standardized patient-based cases for communication training: lessons learned from training residents to communicate diagnostic uncertainty
Source: Adv Simul (Lond). 2021 Jul 22;6:26. doi: 10.1186/s41077-021-00176-y (PMC8296470; doi:10.1186/s41077-021-00176-y)
Supplement: Supplementary file 3 — Additional File 3. Example of an SP Case Summary Guide. [file 41077_2021_176_MOESM3_ESM.docx]

**Additional File 3: Example of an SP Case Summary Guide**

Summary of the Scenario:

You are a 43-year-old male patient with high blood pressure and high cholesterol, and have been in the emergency department all night after coming in late last night for chest pain while climbing up a flight of stairs. The pain was dull and radiated to your right arm. You have never experienced chest pain like this before. You called your wife about these symptoms, and she made you go to the ED.

Your symptoms resolved upon arrival to the ED and they have not returned.

After a series of tests in the ED, you are awaiting on final test called a Cardiac CT to look at your heart more closely.

You feel comforted by the efforts of the treating team, and you have been told everything is normal (apart from the Cardiac CT, the results have not come back yet). You are ready to go home and let your wife know that you didn’t have a heart attack.

Demeanor / Personality and Emotional Starting Point: Reassured

Instructions for the SP, to better comprehend the patient’s demeanor:

| Feelings | REASSURED. The patient is seeking reassurance about not having specific condition (i.e., malignancy) with regards to his chief complaint. He has received this information during the visit.  The patient is receptive and amenable to the conversation. The patient came seeking specific reassurance about something dangerous, and is now comfortable going home without a definite diagnosis. During the conversation, the patient asks reaffirming and clarifying questions throughout the scenario. Example: “*So you are saying that I don’t have anything scary, right*?”).  When/if the physician indicates that no specific diagnosis has been found, the patient responds in a reassured manner. Example: “*I feel so much better knowing this. Thank you*.”). |
| --- | --- |

Instructions for the SP during the conversation:

- Greet the physician upon entry into room.
- Express that you are reassured about how you are feeling physically right now (i.e., your symptoms are better and have not worsened).
- Share that you feel reassured with the ‘normal’ results that have been disclosed with you.
- Ask what the physician sees in your evaluation that can explain your symptoms.
- When/if the physician indicates that no specific diagnosis has been found, you respond that you are nonetheless reassured.
- You can express your reassured state with the following phrases during appropriate parts of the conversation:
  - “So it doesn’t look like anything serious! That’s great news.”
  - “As long as we aren’t finding anything concerning right now, I’m ok.”
  - “Thank goodness. I was worried you’d find something terrible.”

Questions for the SP to ask the physician:

- The goal of these questions is not to prompt a specific checklist item, but rather to provide a prompt for ongoing conversation, when needed.
- SPs should avoid questions that could lead to specific prompting of checklist items.
- Examples include:
  - “So, what is next?”
  - “Should I be concerned about this?”
  - “So what do I tell my family?”

Closing Comment (if needed):

- The closing comment should only be used if the physician is no longer making any progress on the specified checklist and fails to respond to generic prompts, provided above.
- Example: “Thanks for trying to help me today. I think I am fine. I appreciate your time and explanation.”
