# Supplementary material for: Developing standardized patient-based cases for communication training: lessons learned from training residents to communicate diagnostic uncertainty
Source: Adv Simul (Lond). 2021 Jul 22;6:26. doi: 10.1186/s41077-021-00176-y (PMC8296470; doi:10.1186/s41077-021-00176-y)
Supplement: Supplementary file 4 — Additional File 4. Color-Coded Case Template Corresponding to Key Patient Characteristics. [file 41077_2021_176_MOESM4_ESM.docx]

**Additional File 4:**

**Color-Coded Case Template Corresponding to Key Patient Characteristics**

SP Encounter Template

Case Title: Test Case

Case Authors: XCZ, DP, MK, DS

Standardized Patient Name: Jane Doe

Gender: Female

Age Range: 55 y/o

Setting: Emergency Department

Primary vs. Sign-Out Patient: Sign-out

General Appearance / Dress: Lying on stretcher wearing hospital gown

Emotional State: Anxious

Initial Presenting Symptoms: LLQ Abdominal Pain

Opening Statement from the SP after the resident enters the room:

“Hello, doctor. I hope you have some news for me. I have been waiting since the morning. I have been here for hours. I am tired and I am hungry. I’ve had my CT performed, and no one has updated me in the results. Can you please tell me what’s going on!?”

Demeanor / Personality and emotional starting point:

Nervous / Anxious

For the SP, to better comprehend the patient’s demeanor:

| Feelings | Nervous: You were scared to come to the ED for evaluation because your family member recently received a cancer diagnosis in the ED and it took you a lot of courage to come in for evaluation of your abdominal pain.  Anxious: You are worried and nervous about your CT results. A friend of yours was recently diagnosed with colon cancer, and you are terrified about this potential diagnosis. You still do not know the results of the CT, which has been performed hours ago. You fear that the delay may be because of bad news. |
| --- | --- |
| Brief Synopsis | You have been in the ED for several hours for the evaluation of abdominal pain. After finally getting a CT scan, you’re still waiting to hear back from the nurses and the doctor. |

SP Checklist Guide

Instructions for the SP:

You are a 55-year-old female patient with no significant past medical history who presented to the ED for the evaluation of abdominal pain, specifically in your LLQ, which started one day ago. The pain is intermittent, dull, with no radiation. It is sharp in quality. You have not traveled or taken antibiotics. You have no PSH. You have never experienced pain like this in the past. You have no complaints urinating. Your bowel movements are normal. After a lengthy wait upon arrival to the ED, you were seen by the treating team; labs and urine studies were ordered; and you underwent a CT scan with IV and PO contrast media. It has been 2 hours since your CT, and no one has updated you about the results. You are nervous (as your friend was recently diagnosed with colon cancer). You want to know what is causing your pain. You want a diagnosis. You want peace of mind that this is not cancer.

- Greet the provider upon entry into room.
- Express nervousness with regards to the delay.
- Share your anxiety with the ‘negative’ results when they are disclosed to you.
- Ask the provider to tell you what he/she sees on the CT scan that explains your abdominal pain.
- Express anxiety and nervousness when the resident informs you that the CT does not explain the cause of your abdominal pain.
- Ask the resident: “So, what is wrong with me?”
  - State that you are “nervous” when there is no diagnosis disclosed.
  - Question how there can be “no diagnosis.”

Learner Instructions

Patient’s Name: Jane Doe

Age: 50-year-old female

Setting: ED

Initial Chief Complaint on arrival to the ED:

Abdominal pain

Vital Signs over Course of Evaluation:

HR 64 bpm; BP 135/78 mmHg; RR 18; 100% RA; 98.8 F (oral).

VS stable during ED course.

Patient Information Presented at Sign-Out:

55-year-old female with no PMH, who presented to the ED with 1-day Hx of LLQ pain. Patient arrived to the ED with normal vital signs, afebrile, with reproducible tenderness in the LLQ. The remainder of the remainder of exam was unremarkable. Initial plan was to check labs, obtain a CT to evaluate for possible diverticulitis versus any other occult intra-abdominal pathology. She was given Tylenol, which improved her pain, as well as 1 liter of normal saline.

Labs and UA were unremarkable. At the time of sign-out, the CT results are still pending.

ED Course

Most recent vitals: HR 68 bpm; BP 130/74 mmHg; RR 16; 100% RA; 98.8 F (oral).

Repeat exam: no tenderness in the LLQ

Your Task:

1. Approach the patient Jane Doe, and disclose the results of her CT scan.
2. Should the patient have any concerns with these results, discuss them with the patient.
3. Discharge the patient from the Emergency Department.

Results from testing done during the evaluation:

CT Abd/pelvis:

Type of exam: Computed tomography (CT) of the abdomen and pelvis with intravenous and oral contrast.

Clinical history: 55-year-old female with new onset of left lower quadrant pain. Concern for diverticulitis.

Comparison: No previous imaging for comparison.

Technique: 5-mm axial images from the lung bases through the pubic symphysis were acquired following the administration of intravenous and oral contrast. Coronal and sagittal reformatted images were constructed from source data.

Findings:

Lung bases: No pulmonary nodules or evidence of pneumonia.
Cardiac: Base of heart is within normal limits. No pericardial effusion.
Liver: Normal size and contour.

Gallbladder: Normal appearance, no gallstones.
Biliary: No intra or extrahepatic biliary dilation.
Spleen: No splenomegaly.
Pancreas: No mass or ductal dilation.
Kidneys and Adrenals: No masses, stones or hydronephrosis. No adrenal nodules.
Lymph nodes: No lymphadenopathy.
Bowel: No dilation or wall thickening.
Bladder: Within normal limits.
Uterus and Adnexa: The uterus and bilateral ovaries are within normal limits for age.
Bones and soft tissue: There are no osseous or soft tissue abnormalities.
Other: No free fluid within the pelvis.

Impression:

Normal CT of the abdomen and pelvis. No findings on the current CT to explain the patient’s clinical presentation of abdominal pain.
